# Identification of Optimal Decalcification Method and Tissue Preparation Protocol for RNAscope In Situ Hybridization in Rodent Incisor Tooth

**DOI:** 10.3390/dj13110538

**Published:** 2025-11-14

**Authors:** János Konkoly, Árpád Kunka, Attila Szentágotai, Erika Lisztes, Rita Marincsák, Márk Racskó, Judit Bohács, Erika Pintér, Balázs Gaszner, Balázs István Tóth, Viktória Kormos

**Affiliations:** 1 Department of Pharmacology and Pharmacotherapy, University of Pécs Medical School, H-7624 Pécs, Hungary; konkojani1@gmail.com (J.K.); atis585@gmail.com (A.S.); erika.pinter@aok.pte.hu (E.P.); 2 Department of Physiology, Faculty of Medicine, University of Debrecen, H-4012 Debrecen, Hungary; kunka.arpad@dental.unideb.hu (Á.K.); lisztes.erika@med.unideb.hu (E.L.); racsko.mark@med.unideb.hu (M.R.); bohacs.judit@med.unideb.hu (J.B.); toth.istvan.balazs@pte.hu (B.I.T.); 3 Department of Dentoalveolar Surgery, Faculty of Dentistry, University of Debrecen, H-4032 Debrecen, Hungary; 4 Doctoral School of Molecular Medicine, University of Debrecen, H-4032 Debrecen, Hungary; 5 Department of Operative Dentistry and Endodontics, Faculty of Dentistry, University of Debrecen, H-4032 Debrecen, Hungary; marincsak.rita@dental.unideb.hu; 6 Department of Anatomy, Medical School and Research Group for Mood Disorders, Centre for Neuroscience, University of Pécs, H-7624 Pécs, Hungary; balazs.b.gaszner@aok.pte.hu; 7 Institute of Physiology, University of Pécs Medical School, H-7624 Pécs, Hungary

**Keywords:** RNAscope in situ hybridization, decalcification, dental pulp, histology, endodontics

## Abstract

**Background:** RT-qPCR is the gold standard for quantitative gene expression analysis, but it requires homogenized tissue and thus loses spatial information. RNA in situ hybridization (ISH) preserves tissue localization but is technically challenging, especially in calcified tissues such as bone and teeth, where decalcification can damage RNA. RNAscope, an advanced ISH method with high sensitivity and specificity, has been applied successfully to bone, but its use in dental pulp remains largely unexplored despite the pulp’s crucial role in tooth function and health. Our goal was to identify the optimal decalcification process of mouse tooth samples for RNAscope ISH, which preserves RNA integrity in mouse tooth pulp. **Methods:** We tested five different decalcification procedures (EDTA, Plank-Rychlo solution, 5% formic acid, ACD decalcification buffer and Morse solution) on tooth samples from 3-month-old male C57BL/6J mice. Micro-CT and hematoxylin-eosin (HE) staining was performed to evaluate the decalcification, the quality and the microstructure of the sections. RNAscope ISH was used to examine mRNA integrity by analyzing the expression patterns of three housekeeping genes with different expression levels (low, medium and high). **Results:** All five decalcification methods demonstrated well-preserved tissue structure based on HE staining, but RNA integrity was only preserved in the case of mouse dental pulp using the ACD decalcification buffer and Morse’s solution. **Conclusions:** We successfully identified the optimal decalcification procedures preserving RNA integrity in mouse tooth samples, which may be useful for any target RNA examinations by RNAscope ISH in the future.

## 1. Introduction

The gold standard of quantitative gene expression analysis is real-time or quantitative polymerase chain reaction following reverse transcription (RT-qPCR). Although it is a highly sensitive method and provides a huge dynamic range for quantitative detection of virtually any transcripts, it relies on extracted RNA from homogenized tissue sample. Therefore, RT-qPCR studies on small tissue samples comprised by a wide range of cell types (e.g., dental pulp tissue) are seriously limited by the loss of spatial information: the exact cellular origin of the mRNA detected might remain unknown [1]. Conventional RNA in situ hybridization (ISH) is suitable to integrate mRNA-based gene expression and the advantages of histology, preserving the cellular or tissue localization of the targeted transcripts. However, RNA ISH is technically more complex and challenging. To preserve high-quality mRNA suitable for the consecutive ISH, proper sample preparation and tissue fixation procedures play an important role [2,3,4]. This sample processing becomes especially critical if ISH is carried out in calcified hard tissues, like bones or teeth. The appropriate preparation of calcified tissues requires decalcification, applying destructive, mainly acidic chemicals and/or long (>1 week) protocols, which can compromise the quality of the sample by degrading cellular RNA. Therefore, RNA ISH in calcified tissues needs very careful optimization [5,6]. To use this technique in dental samples, even in the case of high expertise with ISH, is very time-consuming and can easily result in improper staining.

RNAscope is an advanced, recently developed ISH technique. Its main innovations are the usage of multiple probes targeting different sequences of the same transcript and an improved signal amplification providing high sensitivity and specificity even if the expression of the targeted transcript is low or the mRNA is partially degraded [7]. It was already successfully applied in decalcified bone samples [8], but hardly any information is available about its application in teeth. The pulp of the tooth is composed of a well-organized spatially strictly arranged cellular architecture, made up of a wide variety of pulpal cells, including odontoblasts, dental fibroblasts, stem cells, various immune cells, as well as vessels and sensory nerve endings. Their coordinated spatially determined local interplay provides a vital contribution to the formation, nutrition, regeneration, sensation and immunological protection of dental tissues [9,10,11]. Therefore, studying the localized gene expression of the pulp is of highest importance for which RNA ISH techniques, like RNAscope ISH, are leading candidate methods. However, the dental pulp is covered by the highly mineralized dentin [12] and in the crown, by the hardest tissue of the body, enamel [13,14], which significantly challenges the application of ISH in the pulp. Though RNAscope technique provides mRNA detection even in decalcified bone samples [15,16,17], the literature indicates that traditional decalcification procedures (e.g., with formic acids or EDTA) may strongly reduce the detectable mRNA transcripts [18,19]. Recent study suggests the cryostat section of undecalcified skeletal tissue to preserve the mRNA integrity; however, one major challenge may be the damage of the morphological structure upon this method [20]. Thus practice-oriented recommendations are highly needed especially for the optimal tissue pretreatment in teeth before RNAscope technique. In the current study, we systematically investigated the compatibility of five decalcification methods with subsequent histological analysis and RNAscope ISH in mouse incisor tooth samples, and we provide an optimal workflow to preserve RNA integrity for the highest quality RNAscope labeling.

## 2. Materials and Methods

### 2.1. Animals

The research was conducted on samples of intact male C57BL6/J mice aged between 12 and 16 weeks. The animals were housed in standard polycarbonate cages (330 mm × 100 mm × 130 mm, 2–5 mice/cage) at the Department of Pharmacology and Pharmacotherapy at the University of Pécs and were provided with standard rodent food (LT/n, Szinbád Kft., Gödöllő, Hungary) and tap water ad libitum at a temperature of 20–24 °C, with a relative humidity of 50–60% and a 12–12 h dark–light cycle. Healthy, untreated mice were subjected to organ and tissue extraction following euthanasia. All procedures were approved by the Animal Welfare Committee at Pécs University, National Scientific Ethical Committee on Animal Experimentation in Hungary (BA/73/00064-4/2025) in agreement with the directive of the European Communities Council in 1986, and with the Law of XXCIII, in 1998, on Animal Care and Use in Hungary. We have consistently striven to minimize the number of animals used in our studies and to limit their suffering to the greatest extent possible.

### 2.2. Sample Collection

A total of 20 maxillary and mandibular incisors and molars were obtained from 20 male C57BL/6J mice aged between 12 and 16 weeks. First, deep anesthesia was induced with intraperitoneally administered urethane (2.4 g/kg) (Sigma-Aldrich, St-Louis, MO, USA; Cat. No.: 501838936). Mice were then transcardially perfused with 20 mL of ice-cold 0.1 mol/L phosphate-buffered saline (PBS, pH 7.4), followed by 150 mL of 4% paraformaldehyde (PFA) (Sigma-Aldrich; Cat. No.: 158127) solution. Both the upper and lower incisors with the surrounding alveolar tissues and gingiva were postfixed in PFA solution at 4 °C for a period of three days. Subsequently, samples were randomly assigned into five groups (n = 4 per group) and each underwent distinct decalcification procedures.

### 2.3. Decalcification Procedures

The efficacy of five distinct decalcification methods was evaluated. The detailed composition of the applied solutions and the conditions of the incubations are presented in Table 1. All components were purchased from Sigma-Aldrich. 50 mL of decalcifying solution was added to each sample. In each instance, the effectiveness of decalcification was assessed manually, and the dehydration process was started upon the attainment of the elastic properties of the samples.

### 2.4. Micro-CT

Five distinct decalcification protocols were applied to mouse tooth samples, and their effectiveness was quantitatively evaluated by mineral density measurements using micro-CT on molar teeth from the same animals, from which we measured RNA integrity in incisor teeth. The images were taken with a Skyscan 1176 micro-CT device marketed by Bruker (Billerica, MA, USA). The voltage was 30 kV, the current was 360 µA and the exposure time was 3500 ms long during every scan with a 0.5 mm aluminum filter in use and with 0.7 degree increments in a 180 degree rotation. One scan took approximately 36 min. The side of one pixel corresponded to 8.74355 µm in reality. The raw images were reconstructed with NRecon (v.: 1.7.4.2) software. These selected areas were analyzed using CTan (v.: 1.20.8.0+) and Image J (version 1.52a, NIH, USA) software. Before evaluation, the software was calibrated to measure dental density.

### 2.5. Sample Preparation and Sectioning

Following dehydration, the samples were oriented and embedded in paraffin. Cross-sectional slices of the teeth, 5 µm in thickness, were prepared using a microtome (HM 430, Thermo Fisher Scientific, Waltham, MA, USA). The slices were mounted on SuperFrost Ultra Plus slides (Thermo Fisher Scientific; Cat. No.: 1014356190). No samples failed during embedding or RNAscope processing.

### 2.6. Hematoxylin-Eosin Staining

Hematoxylin-eosin (HE) staining was conducted on the tooth sections to ascertain the efficacy of the decalcification procedures and to evaluate the integrity of the histological microstructure. During the staining process, the sections were treated with three 5 min applications of xylene (Sigma-Aldrich; Cat. No.: X4126) and two 3 min applications of isopropyl alcohol (Sigma-Aldrich; Cat. No.: I9516). Subsequently, the specimens were treated with Mayer’s hematoxylin (BioGnost, Zagreb, Croatia; Cat. No.: HEMM-OT-X) for a period of five minutes, after which they were blued in tap water for a further five minutes. The sections were treated with eosin (BioGnost; Cat. No.: EOY-10-OT-X) for a period of two minutes, rinsed with distilled water and then treated with two further applications of isopropyl alcohol for three minutes each. The slides were placed in xylene for a period of two minutes and then covered with DPX mountant (Fluka/Honeywell, Seelze, Germany).

### 2.7. RNAscope In Situ Hybridization

RNAscope ISH was used to investigate RNA integrity in mouse paraffin-embedded tooth samples. Pre-treatment was performed according to the RNAscope Multiplex Fluorescent Reagent Kit v2 user manual (ACD, Hayward, CA, USA; Cat. No. 323100). Sections were incubated at 60 °C for 1 h, then deparaffinized in xylene for 2 × 5 min, followed by 2 × 2 min absolute ethanol treatment. The sections were then dried at 60 °C for 5 min, followed by treatment with hydrogen peroxide for 10 min to inhibit endogenous peroxidase, followed by Milli-Q (MQ) aqueous washing. Three different permeabilization methods were then tested for optimization:

Permeabilization method 1. Sections were incubated in 20 mg/mL proteinase K (Sigma-Aldrich; Cat. No.: P2308) solution pre-warmed to 37 °C for 20 min in a 37 °C incubator.

Permeabilization method 2. Sections were treated in a microwave oven at the 700 W setting for 10 min in 100 mL citrate buffer pH 6 (Sigma-Aldrich; Cat. No.: C9999). After MQ water washing, 3 min absolute ethanol treatment was followed by 5 min drying at 60 °C. After circumscribing with a hydrophobic pen (Vector Immedge Pen, Vector Laboratories, Newark, CA, USA), sections were treated with Protease Plus (ACD; Cat. No.: 322331) for 30 min at 40 °C in a humidified steam chamber.

Permeabilization method 3. Sections were boiled at high pressure for 10 min in Target Retrieval solution (ACD; Cat. No.: 322000). After MQ water washing, 3 min absolute ethanol treatment was followed by 5 min drying at 60 °C. After circumscribing with a hydrophobic pen (Vector Immedge Pen), sections were treated with Protease Plus (ACD; Cat. No.: 322331) for 30 min at 40 °C in a humidified steam chamber.

Following the completion of the explorations, the subsequent steps were identical for all samples and followed the instructions set forth in the user manual of the manufacturer (ACD). Briefly, after permeabilization, the sections were washed with MQ water and then hybridized for a period of two hours with the mouse 3-plex positive control probes (ACD, Cat. No.: 320881). This reagent targets three distinct mouse housekeeping gene-specific sequences, expressed at different levels and stained with different fluorochromes. Low expression RNA polymerase II A subunit (*Polr2a*) mRNA was stained with fluorescein, medium expression peptidyl-prolyl cis-trans isomerase B (*Ppib*) mRNA was visualized with cyanine 3 (Cy3), while highly expressed ubiquitin C (*Ubc*) mRNA was labeled with cyanine 5 (Cy5). As negative control, 3-plex negative control probe was used targeting the bacterial L-2,3-dihydrodipicolinate reductase mRNA (*dabP*). Following the hybridization, signal amplification and channel development steps were performed following the manufacturer’s instructions (ACD).

Finally, samples were washed 2 × 15 min in PBS and were stained with 4′,6-diamidino-2-phenylindole (DAPI) (ACD; Cat. No.: 320858) in order to mark the nuclei. Following the washing process, the slides were covered with ProLong Gold Antifade (Thermo Fisher Scientific; Cat. No.: P36930) medium and stored at −20 °C until imaging.

### 2.8. Microscopy, Morphometry

HE sections were examined using a Nikon Microphot FXA microscope and captured using a Spot RT color digital camera (Nikon, Tokyo, Japan). RNAscope ISH stained samples were digitalized using an Olympus FluoView 1000 confocal microscope (Olympus, Hamburg, Germany) in analog mode with sequential scanning. Fluorescence imaging was conducted with the following settings: confocal aperture of 80 µm, optical thickness of 3.5 µm, resolution of 1024 × 1024 pixel, using 40× and 60× objectives. The excitation and emission spectra of fluorescent dyes were selected using FluoView software (FV10-ASW; Version 0102, Olympus) and illuminated with 405, 488, 550 and 647 nm laser beams to excite DAPI, Fluorescein, Cy3 and Cy5, respectively. The sections were scanned in four channels and digitally merged subsequently. In the digital images, the colors blue (DAPI), green (Fluorescein), red (Cy3) and white (Cy5) represent the four different channels.

Densitometry was performed using ImageJ software (version 1.52a, NIH, USA) on non-edited pictures to quantify the RNAscope signals for each housekeeping gene across the different decalcification methods. Intensity of the fluorescence was measured in four manually selected surface areas in the dental pulp using non-edited images The signal density was corrected for the background signal. The average of the specific signal density (SSD) of areas was determined in four sections per animal. The average of these four values represented the SSD value of one mouse. The SSD was expressed in arbitrary units (a.u.).

### 2.9. Statistical Analysis

Statistical analysis was performed using Statistica 13.5.0 software. Data are presented as mean ± SEM. All datasets were tested for normal distribution (Kolmogorov–Smirnov) and homogeneity of variance (Levene). The main effects were studied by one-way analysis of variance (ANOVA, variable: decalcification procedure) followed by Tukey’s post hoc test. Results with a *p*-value lower than 0.05 were considered statistically significant. Minor variability between animals was observed within the group but without clear outliers.

## 3. Results

The objective of our study was to optimize a decalcification and sample preparation procedure for histological examination of teeth, which allows proper sectioning of the teeth without damaging the mRNA and is therefore suitable for RNAscope ISH staining. Five different decalcification methods were tested, then micro-CT and HE staining were performed to check decalcification and the microstructure of the samples, respectively. The RNA integrity was investigated by using mouse 3-plex positive control probes to detect genes with high, medium and low expression simultaneously.

### 3.1. Effect of Decalcification on the Density of the Teeth

**Figure 1:**
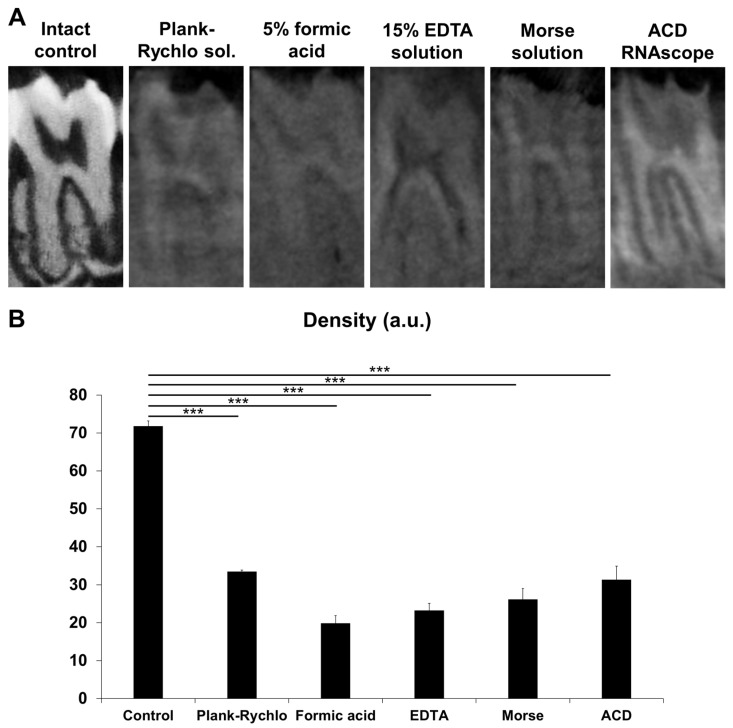
Effect of decalcification on the density of mouse teeth. Representative micro-CT images of mouse tooth specimens following decalcification using Plank-Rychlo solution, 5% formic acid, ethylenediaminetetraacetic acid (EDTA), Morse solution and ACD’s Bone Decalcification Buffer (**A**). Statistical evaluation of the specific signal density (**B**). Columns show means ± SEM of the density (n = 4; *** *p* < 0.0001; Tukey’s post hoc test upon one-way ANOVA).

Five distinct decalcification protocols were applied to mouse tooth samples, and their effectiveness was quantitatively evaluated using micro-CT on molar teeth from the same animals. Mineral density measurements revealed a significant reduction in density across all decalcified samples compared to the untreated, intact control group. One-way ANOVA confirmed a highly significant overall difference among the groups (*p* = 3.06 × 10^−11^), and subsequent post hoc comparisons indicated that each decalcification treatment resulted in significantly lower density values than the controls (*p* < 0.0001). These findings demonstrate that all five decalcification protocols effectively removed calcium from the dental tissues (Figure 1).

### 3.2. Effect of Decalcification on the Histological Structure of the Teeth

In general, all five methods resulted in satisfying decalcification, and the samples could be sectioned without any obvious difficulties. However, moderate differences were detected in the quality of the histological structure and the length of the methods differed a lot.

**Figure 2:**
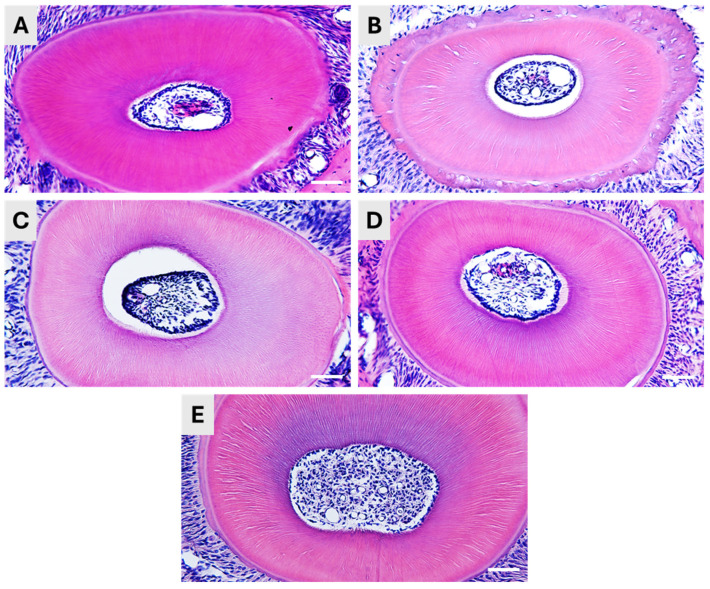
Effect of decalcification on the histological structure of mouse teeth. Representative microscopic images of hematoxylin-eosin stained mouse tooth specimens following decalcification using (**A**) Plank-Rychlo solution, (**B**) 5% formic acid, (**C**) ethylenediaminetetraacetic acid (EDTA), (**D**) Morse solution and (**E**) ACD’s Bone Decalcification Buffer. Scale bar: 100 µm.

The Plank-Rychlo decalcifying solution contains strong inorganic acid (hydrochloric acid), as well as organic formic acid. After decalcifying in this solution, the tooth samples were found well-sectionable, and the HE staining displayed a preserved pulpal structure (Figure 2A). The method’s advantage is the relatively short incubation time, only 6 h at 4 °C.

If we applied only organic formic acid in 5% concentration, the required decalcification time was much longer, as one week at 4 °C was needed to reach the consistency ideal for sectioning. The histological structure of the teeth was well-preserved, as indicated by the HE staining. However, we observed some shrinkage of the pulp tissue (Figure 2B).

Applying EDTA solution is a generally suggested method for decalcification if destructive acids are to be avoided. In our hands, incubation in 15% EDTA solution at 56 °C for a week resulted in soft, flexible and excellent-to-cut samples. The excellent decalcification was also indicated by the proper retrieval of the dentinal canals, as shown by the HE staining (Figure 2C). Although the hard tissue was properly processed, we experienced a decrease in the pulp volume, and it was detached from the dentin, as it was also seen in the case of formic acid decalcification.

The application of Morse solution was optimal for fast dental decalcification. The application of the mixture of 10% citrate and 20% formic acid for 12 h resulted in a fast and proper decalcification and in excellently sectionable tissues. The histological structure of the teeth was well-preserved in both the hard tissue (dentin) and the pulp of the teeth (Figure 2D).

Finally, we have applied a decalcifying solution of the ACD suggested for decalcification of bone samples prior to RNAscope. Our tooth samples have been incubated for two weeks at 4 °C to reach a soft, well-sectionable consistency, according to the ACD protocol. The histological structure was intact after the decalcification (Figure 2E).

### 3.3. Optimalization of the Permeabilization and Effect of Various Decalcification Methods on the RNA Integrity

Once we were satisfied that all decalcification procedures were successful in terms of sectioning and microstructure, we performed RNAscope ISH to test for the preservation of RNA integrity. First, we tested 3 different permeabilization methods for the retrieval of the RNA. Digestion with proteinase K (Permeabilization method (1) was found to be ineffective, as the ISH probes did not penetrate into the samples properly and resulted in weak, uncertain staining. Incubation in a citrate solution in a microwave oven (Permeabilization method (2)) weakened the adhesion; therefore, several samples floated off the slides after the treatment. The most optimal digestion method was the treatment in Target Retrieval solution (ACD) in a streamer for 10 min followed by Protease Plus (ACD) treatment for 30 min at 40 °C (Permeabilization method (3)). Mouse 3-plex positive control probes specific to 3 mouse housekeeping genes were used during ISH, providing adequate information about the RNA integrity. Samples prepared by the five different decalcification procedures discussed previously were tested.

**Figure 3:**
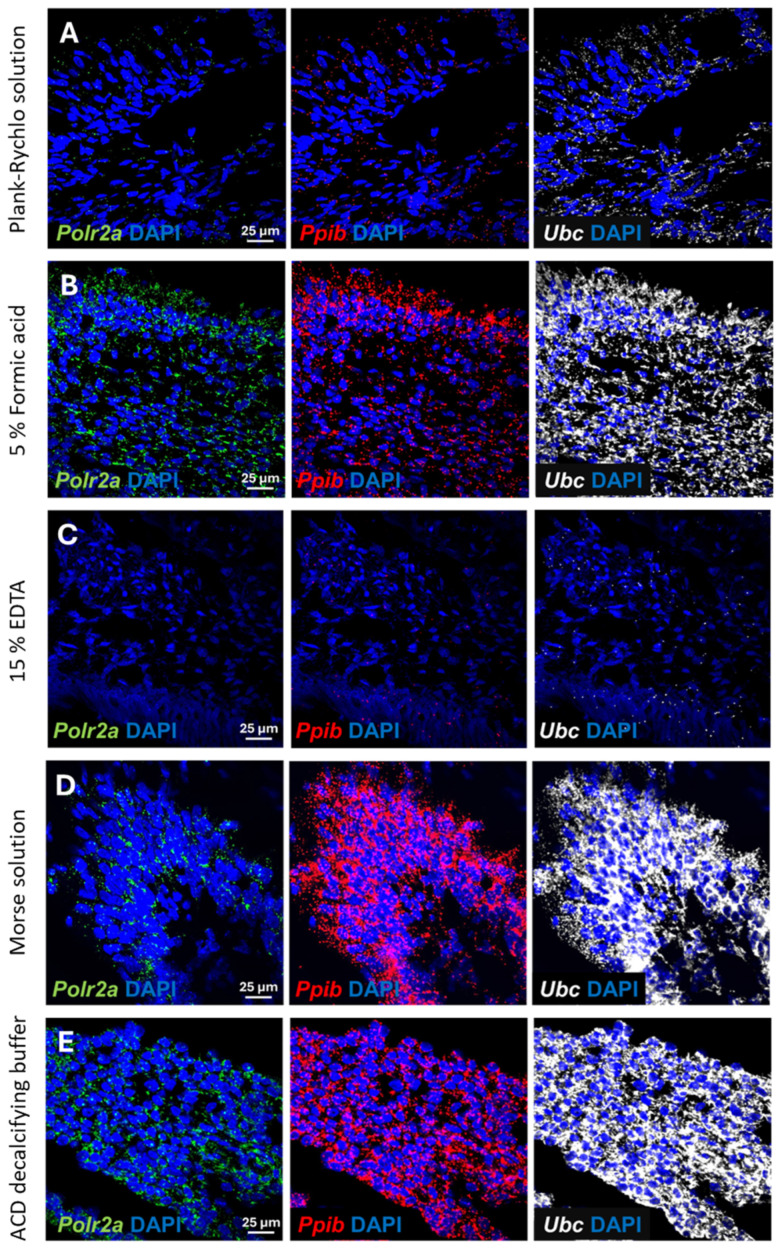
Effect of decalcification on the RNA integrity in mouse teeth. Representative fluorescent images of RNAscope in situ hybridization using 3-plex positive control probes on mouse tooth samples after decalcification with (**A**) Plank-Rychlo solution, (**B**) 5% formic acid, (**C**) ethylenediaminetetraacetic acid (EDTA), (**D**) Morse solution and (**E**) ACD’s Bone Decalcification Buffer. Expression of RNA polymerase II A subunit (*Polr2a*; green), peptidylprolyl isomerase B (*Ppib*; red) and ubiquitin C (*Ubc*; white) housekeeping genes which are expressed in low, medium and high copy number, respectively, in mouse samples. The relative staining intensity of each target reflects the integrity of the mRNA transcripts in the sample. Nuclear counterstaining was performed with 4′,6-diamidino-2-phenylindole (DAPI, blue). Scale bar: 25 µm.

Decalcification in Plank-Rychlo solution resulted in strong degradation of the RNA. All three examined housekeeping genes were hardly detectable, although we could still recognize the expected signal intensity difference between the three housekeeping genes (Figure 3A).

Treatment with 5% formic acid for one week resulted in a somewhat better RNA integrity. All housekeeping genes were clearly detectable by RNAscope ISH, although their level was lower than expected, indicating a partial RNA degradation in the samples (Figure 3B).

The EDTA solution, which is the most commonly used decalcification method to preserve sensitive, degradable protein antigens, caused a massive mRNA destruction and resulted in the worst RNAscope signal among the investigated methods. Consequently, the *Polr2a* transcripts, characterized by low expression, became undetectable. The midlevel-expressing *Ppib* transcripts were also barely detectable. Even the probes targeting the high-copy *Ubc* transcripts resulted only in a very weak ISH signal (Figure 3C).

In contrast to the previous methods, decalcification in Morse solution or in the ACD’s Bone Decalcification Buffer resulted in high-quality ISH signals (Figure 3D,E). Even the low-copy number *Polr2a* transcripts were clearly detectable with high confidence, whereas the *Ppib* and *Ubc* targeting probes resulted in very strong punctate and confluent signal, in line with their expected expression level.

The above results indicated that decalcification with ACD’s Bone Decalcification Buffer or Morse solution preserved the RNA integrity that was highly suitable for a subsequent RNAscope ISH.

**Figure 4:**
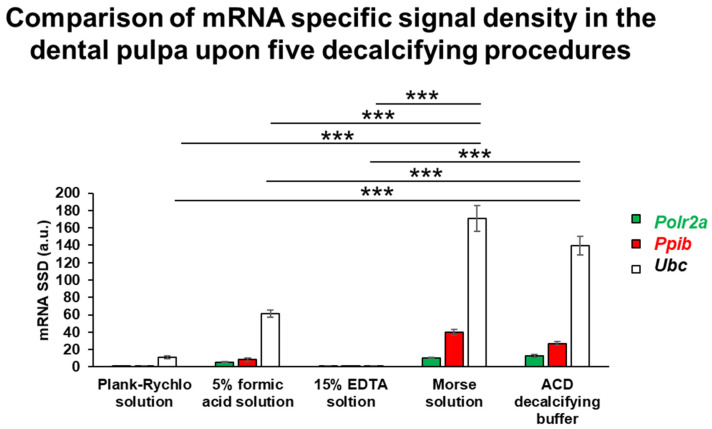
Histogram provides a comparison of mRNA signals upon five different decalcification procedures. Specific signal densities of mRNAs of RNA polymerase II subunit A (*Polr2a*, green bars), peptidyl-prolyl cis-trans isomerase B (*Ppib*, red) and ubiquitin C (*Ubc*, white) were measured in the dental pulp expressed in arbitrary units (a.u.). Plank-Rychlo solution, 5% formic acid solution and 15% EDTA solution resulted in low or undetectable mRNA signal that affected all three examined transcripts as their signal density was significantly lower than measured in preparations decalcified by Morse solution or in ACD decalcifying buffer. The latter two solutions preserved the sensitivity of the technique for low (*Polr2a*), mid (*Ppib*) and high copy (*Ubc*) mRNAs. The comparison revealed that Morse solution and ACD decalcifying buffer did not differ statistically from each other. *** *p* < 0.001, compared to the specific signal density of the same mRNA, according to Tukey’s post hoc test upon one-way ANOVA (for main effect of desalination method), (n = 4).

RNA integrity was assessed following the five different decalcification protocols using RNAscope ISH combined with densitometric quantification of three housekeeping genes exhibiting distinct expression levels. This analysis aimed to determine which decalcification method best preserved RNA quality. One-way ANOVA revealed a statistically significant difference among the groups (*p* = 6.25 × 10^−13^). Post hoc comparisons showed that samples decalcified with Morse solution and ACD solution maintained the highest RNA integrity, with no significant difference between these two treatments. In contrast, all other decalcification protocols resulted in a significantly reduced RNA signal intensity compared to both Morse and ACD groups (*p* < 0.0001). These findings indicate that Morse and ACD solutions most effectively preserve RNA integrity during tooth sample decalcification (Figure 4).

## 4. Discussion

Although several protocols are set for decalcification and proper histological preparation, especially for bones, their suitability for consequent RNA ISH is less investigated [18,19], and we have only limited information, especially about the tooth as histological specimen. In our study, we systematically tested and evaluated five decalcification methods for RNAscope ISH on mouse incisor samples. The techniques used in this research were optimized originally for bone tissue; thus, our present aim was to clarify the effect of these various decalcification procedures on the mRNA detectability in mouse dental pulp.

RNAscope is a novel RNA ISH technique which opens new avenues in the qualitative and quantitative in situ gene expression studies. It is suitable to detect virtually any transcripts, even in calcified tissues, at high specificity and superior sensitivity [15,16,17,20]; therefore, it provides a highly competitive alternative to immunohistochemistry in several situations [3,7]. However, its application is especially challenging in the teeth.

Decalcifying dental tissue is an essential step for histological examination. Without removing fluor- and hydroxyapatite crystals and other calcium salts, microtome blades cannot cut the tissue, the blades would be damaged and the specimen would shatter. Upon decalcification the tissue gets soft enough to obtain thin, uniform sections for microscopic analysis. Proper decalcification removes minerals without damaging soft tissue elements, allowing clear microscopic visualization of cells, fibers and extracellular matrix structures and for this particular purpose retains RNA integrity. Calcium deposits also interfere with routine histological stains (e.g., HE, trichrome) while decalcification eliminates these mineral barriers, ensuring even dye penetration and accurate staining. Inadequate or uneven decalcification can lead to tearing, folding, or poor staining of sections. On the other hand, over-decalcification, may damage tissue proteins, mRNAs and distort morphology, so timing and reagent choice are critical [5,6].

There are several methods available for decalcification, and the vast majority of them can be classified into three groups based on the agent used to remove the mineral deposits. Minerals can be solved out by (1) strong inorganic acids which, in general, provide an excellent removal of the inorganic components but are highly corrosive and easily harm vulnerable biomolecules like protein antigens or RNA. (2) The use of weaker organic acids, like formic acid, offers a gentler option and may more effectively preserve organic macromolecules while showing with similar effectiveness in removal of extracellularly deposited inorganic salts. Alternatively, the use of (3) chelating agents, such as EDTA, is recommended to preserve highly vulnerable antigens for immunohistological detection [21,22].

In this study, we investigated five decalcification methods using mouse incisor samples and compared their effect on the histological microstructure and suitability for RNAscope ISH. The decalcification protocols are summarized in Table 2.

The Plank-Rychlo solution contains both a strong inorganic (hydrochloric acid) and an organic (formic acid) acid, as well as aluminum chloride. It is recommended for rapid bone decalcification. In our samples, its application for a short incubation period indeed resulted in an excellent decalcification; at the same time, it excellently preserved the morphological structure. However, it deteriorated the RNA integrity, indicated by very weak RNAscope signals. These results are in good accordance with earlier data on bone decalcification. Shibata et al. [22] described in mouse mandibles that the Plank-Rychlo solution and other inorganic acid-based decalcifying agents, although excellently preserving the morphology of the ameloblast layer, dramatically decreased both 28s rRNA and osteoblast-specific mRNA signals in conventional ISH.

Formic acid is frequently used for decalcifying bone tissue and in comparison with inorganic acids [21] it is considered as a gentler method offering better antigen preservation for immunohistochemistry. In our hands, formic acid resulted in an ideal preservation of dental histomorphology, although we observed some shrinkage of the pulp tissue. Its disadvantage was the longer incubation time in comparison with the Plank-Rychlo solution. Of note, the RNA integrity was better than observed in samples treated with the inorganic acid solution; the relatively weak ISH signal indicated partial mRNA degradation. Similar results were reported earlier in rat and mouse mandibles: formic acid decalcification had slightly compromised the quality of histomorphology due to some vacuolization in the tissues [22,23]. However, the integrity of 28S rRNA was relatively well preserved [22].

EDTA is considered as the gold standard of decalcification if a gentle procedure is needed. For instance, it is ideal if the antigens to be detected are sensitive for acidic degradation [18,19,21,24]. In our experiment it indeed ideally preserved the histomorphology. Nevertheless, the use of EDTA resulted in a serious degradation of mRNA, since we observed hardly any RNAscope ISH signal. Here, it is important to mention that the largely different incubation conditions significantly interfere with mRNA quality. For instance, it is well known that high temperature or longer incubation time can negatively affect RNA integrity. In our protocol, we incubated the samples in EDTA solution at 56 °C to accelerate the decalcification process. Despite this, one week was required to achieve the properly sectionable consistency of the incisors. In an earlier study, elevating the temperature of the decalcifying EDTA solution from room temperature just to 37 °C reduced the incubation time requirement of complete decalcification in rat mandible samples to half. However, this higher temperature, despite the shorter incubation time, resulted in a damaged cellular morphology [23]. In contrast, in mouse mandibles, both rRNAs and mRNAs were preserved upon decalcification in EDTA solution [22]; however, the temperature condition of incubation was not reported in the study. Beyond temperature and incubation time, the efficacy of EDTA-based decalcification is also crucially influenced by the pH of the solution. In an acidic environment, the EDTA did not cause any decalcification, while it provided an ideal effect at neutral pH, and the alkalic pH of the solution even further shortened the necessary incubation time [21].

In our hands, the ACD decalcifying buffer for bone used for 2 weeks at 4 °C resulted in an excellent decalcification without compromising the tissue structure quality, in combination with outstanding preservation of RNA integrity of the tooth samples. The Morse solution provided similar high-quality results. Of note, this was achieved upon a much shorter incubation period of only 12 h. This is in agreement with earlier studies, where the Morse solution was found to be an excellent decalcifying solution, comparable with EDTA in both rodent and human dental tissue [25,26]. Morse solution was also found to provide preserved tissue morphology in decalcified cochlear samples accompanied by excellent mRNA quality as shown in qPCR tests [27]. Therefore, based both on our present results and earlier observations we recommend both the ACD buffer and Morse solution for decalcification of tooth samples to be subjected to RNAscope ISH.

In clinical cases, immunohistochemical procedures are performed most frequently on pulp tissue removed from the tooth. Currently, the use of the RNAscope ISH technique on human pulp is not yet a routine examination method, but due to its advantages, it is expected to become increasingly widespread. Until today, only few studies reported the application of RNAscope to detect long non-coding RNA [28] or viral RNA [29] in human dental pulp tissue. Importantly, in these projects extirped pulp tissue was subjected to RNAscope ISH, which largely limits the quality and reliable assessability of pulp tissue components. Sectioning of the whole tooth including both the hard tissues together with the soft contents of the pulp cavity may allow a more accurate localization of mRNAs in teeth.

In summary, the present study was designed to identify the optimal method which allowed consistent comparisons across multiple decalcification protocols capable for RNAscope technique in the dental pulp of mouse incisors. As for limitations, it should be noted that (i) differences in temperature and time used in the diverse protocols may contribute to the variability in the morphological structure and RNA integrity [23,30,31,32], and (ii) the restriction to incisor samples and the single animal model constrain the generalizability of the research. Thus, future studies should focus on extending these findings to molars and to distinct animal species.

## 5. Conclusions

In this study, we identified and optimized reliable decalcification procedures tailored for RNAscope ISH in dental tissues preserving both tissue histomorphology and RNA integrity. Each procedure tested in the present study provided satisfactorily decalcified, well-sectionable tooth samples. However, the moderate-strong degradation of mRNA and the shrinkage of pulpal tissue limit the applicability of Plank-Rychlo solution, formic acid or EDTA in RNA-based techniques. In contrast, the well-preserved tissue structure and the intact mRNA integrity observed after Morse solution and ACD buffer treatment indicate that both techniques may provide optimal decalcification method for future RNAscope studies in dental pulp.

## Figures and Tables

**Table 1:** Decalcification solutions and protocols. ACD: Advanced Cell Diagnostics Company; EDTA: ethylenediaminetetraacetic acid.

Decalcifying Agents	Composition of Decalcifying Solution	Conditions for Decalcification
**Plank-Rychlo solution**	8.5 mL hydrochloric acid (Cat. No.: 320331), 5 mL formic acid, 7 g aluminum chloride (Cat. No.: 237051), 100 mL distilled water	6 h at 4 °C
**Morse solution**	10% sodium citrate (Cat. No.: S4641), 20% formic acid in distilled water	12 h at 4 °C
**5% formic acid** (Cat. No.: F0507)	5% formic acid in distilled water	1 week at 4 °C
**EDTA** (Cat. No.: E9884)	150 g EDTA, heated in 1000 mL distilled water until clear, followed by 15 g sodium hydroxide (Cat. No.: 221465) (pH 7–7.4)	1 week at 56 °C
**ACD Bone Decalcification Buffer** (Cat. No.: 321918)	Ready to use formulation. The company treats the composition of the solution as a trade secret	2 weeks at 4 °C

**Table 2:** Summary of the results. ACD: Advanced Cell Diagnostics Company; EDTA: ethylenediaminetetraacetic acid.

Decalcifying Agents	Histological Structure	mRNA Integrity
**Plank-Rychlo solution**	Well-preserved structure in both the dentin and the pulp	Strong degradation
**5% formic acid**	Well-preserved structure in the dentin with a weak shrinkage of the pulp	Partial degradation
**EDTA**	Well-preserved structure in the dentin with a weak shrinkage of the pulp	Almost complete degradation
**Morse solution**	Well-preserved structure in both the dentin and the pulp	Preserved mRNA integrity
**ACD Bone Decalcification Buffer**	Intact structure in both the dentin and the pulp	Preserved mRNA integrity

## Data Availability

The datasets used and/or analyzed during the current study are available from the corresponding author upon reasonable request.

## Abbreviations

The following abbreviations are used in this manuscript:

ISH	In situ hybridization
HE	Hematoxylin-eosin
RT-qPCR	Real-time quantitative polymerase chain reaction
PBS	Phosphate-buffered saline
PFA	Paraformaldehyde
MQ	Milli-Q
*Polr2a*	RNA polymerase II A subunit
*Ppib*	Peptidyl-prolyl cis-trans isomerase B
Cy3	Cyanine 3
*Ubc*	Ubiquitin C
Cy5	Cyanine 5
*DabP*	L-2,3-dihydrodipicolinate reductase mRNA
DAPI	4′,6-diamidino-2-phenylindole
EDTA	Ethylenediaminetetraacetic acid
